# Plasmaphosphorylated tau as biomarkers for multiple sclerosis diagnosis, subtyping, and prognosis

**DOI:** 10.1093/braincomms/fcaf510

**Published:** 2026-01-02

**Authors:** Chen Hu, Xuemei Zeng, Lili Zhang, Anuradha Sehrawat, Megan Powell, Emily Song, Elizabeth L S Walker, Alexis Watterson, Wen Zhu, Thomas K Karikari, Zongqi Xia

**Affiliations:** Department of Epidemiology, University of Pittsburgh, Pittsburgh, PA 15260, USA; Department of Psychiatry, University of Pittsburgh, Pittsburgh, PA 15260, USA; Department of Neurology, University of Pittsburgh, Pittsburgh, PA 15260, USA; Department of Psychiatry, University of Pittsburgh, Pittsburgh, PA 15260, USA; Department of Neurology, University of Pittsburgh, Pittsburgh, PA 15260, USA; Department of Neurology, University of Pittsburgh, Pittsburgh, PA 15260, USA; Department of Neurology, University of Pittsburgh, Pittsburgh, PA 15260, USA; Department of Neurology, University of Pittsburgh, Pittsburgh, PA 15260, USA; Department of Neurology, University of Pittsburgh, Pittsburgh, PA 15260, USA; Department of Psychiatry, University of Pittsburgh, Pittsburgh, PA 15260, USA; Department of Neurology, University of Pittsburgh, Pittsburgh, PA 15260, USA

**Keywords:** multiple sclerosis, biomarkers, tau phosphorylation, prognosis, neurodegeneration

## Abstract

Blood-based biomarkers are crucial for individualized management of multiple sclerosis (MS). Blood neurofilament light chain (NfL) and glial fibrillary acidic protein (GFAP) have shown promising clinical utility in MS, but they are insufficient to guide clinical management. Plasma tau proteins remain underexplored despite the growing evidence of shared pathology in Alzheimer’s disease and MS. We aimed to: (i) assess the utility of plasma tau biomarkers [phosphorylated tau 181 (p-tau181), p-tau217 and total tau (t-tau)] in MS diagnosis, subtyping and prognosis; and (ii) compare their performance with NfL and GFAP. From a clinic-based prospective cohort, we evaluated 160 people with MS [pwMS; 117 with relapsing–remitting MS, 43 with progressive MS (PMS)] and 20 non-MS controls, all with baseline plasma samples. We measured baseline plasma concentrations of p-tau181, p-tau217, t-tau, NfL and GFAP using ultrasensitive immunoassays. We collected demographics, clinical information, and longitudinal multi-modal outcomes (Patient Determined Disease Steps, normalized age-related MS severity score, walking speed, manual dexterity, cognitive performance, retinal nerve fibre layer thickness, total brain volume and grey matter volume) over a median follow-up of 3.0 years (interquartile range, 3.5). Adjusting for demographic and clinical covariates, we evaluated associations between biomarkers and MS diagnosis, subtypes, and prognosis. We examined the enhanced value of tau markers, in addition to NfL and GFAP, for subtype distinction and outcome prediction. Participants were enrolled between 2017 and 2023. Assays were performed in August 2023. Analyses were conducted in December 2024. Participants (*n* = 180) had a median age of 51 years and were predominantly women (68%) and non-Hispanic white (91%). Compared with controls, pwMS had higher levels of p-tau217 (1.0 versus 0.7 pg/ml; *P* = 0.04) and NfL (14.1 versus 9.0 pg/ml; *P* < 0.01). Among pwMS, higher p-tau181 (adjusted odds ratio (aOR) [95% confidence interval (CI)] = 2.3 [1.4, 4.1]) and p-tau217 (aOR [95% CI] = 3.0 [1.8, 5.7]) were associated with PMS. These markers improved MS subtype classification accuracy beyond clinical features, NfL and GFAP. Higher baseline p-tau181 and p-tau217 predicted worse disability, functional outcomes and imaging outcomes independent of other biomarkers. Plasma p-tau181 and p-tau217 are promising biomarkers for MS subtype classification and disability prediction, providing complementary information to NfL and GFAP. Further studies to validate their potential clinical utility in guiding MS management are warranted.

## Teaser

Hu et al. report that plasma phosphorylated tau (181 and 217) proteins distinguish progressive from relapsing multiple sclerosis and predict future disease progression. Plasma phosphorylated tau proteins may have clinical utility in complementing other existing blood biomarkers for disease monitoring and prognostication and potentially in guiding multiple sclerosis care.

## Graphical Abstract

**Graphical Abstract:**
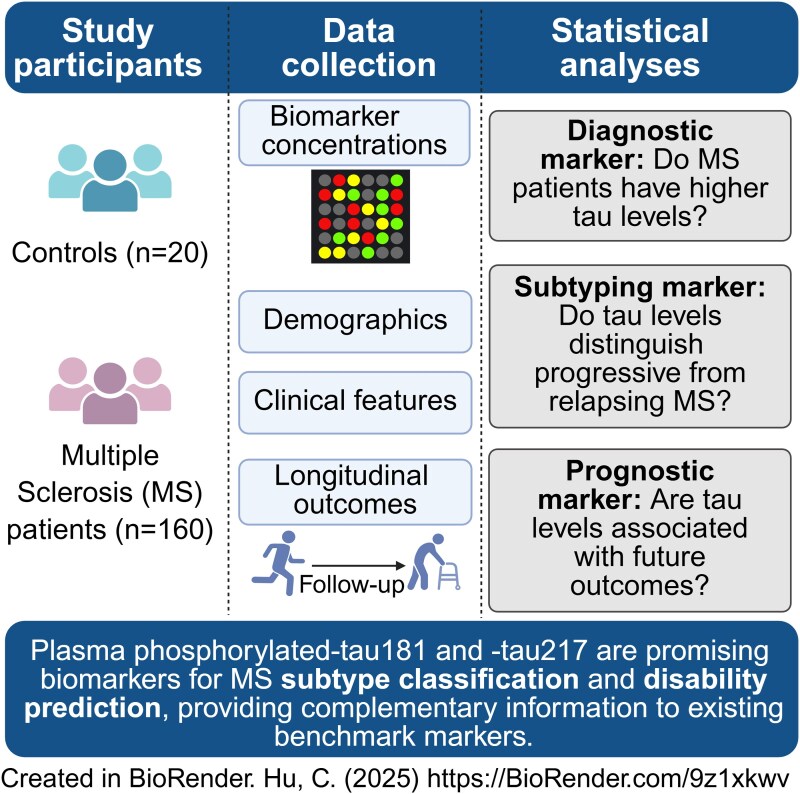

## Introduction

Multiple sclerosis (MS) is a chronic disease of the central nervous system (CNS) causing progressive accumulation of neurological disability.^1,2^ Based on clinical course, MS subtypes include relapsing–remitting (RRMS), primary progressive (PPMS), or secondary progressive (SPMS), which inform disease-modifying therapy (DMT) selection.^3^ Complex pathophysiological mechanisms drive variable disease progression trajectories among people with MS (pwMS).^4,5^ Fluid biomarkers offer promise to guide individualized management.^6–8^ Cerebrospinal fluid (CSF) biomarkers have limited clinical feasibility.^9–11^ In contrast, blood biomarkers are less invasive to collect, more reproducible to assay, and better suited for longitudinal monitoring in chronic neurological disorders. Recent advances in assay development have expanded the availability and reliability of blood-based biomarkers, providing opportunities to investigate their potential utility in MS.^12,13^

Blood neurofilament light chain (NfL) and glial fibrillary acidic protein (GFAP) disease have shown promise to inform MS disease activity and progression.^14–18^ However, their capabilities to diagnose MS and differentiate MS subtypes are limited.^19^ In current practice, MS subtypes are retrospectively confirmed using clinical history and MRI. This limitation delays the recognition of patients with progressive subtypes and hinders timely clinical decisions given the limited DMT options for progressive MS (PMS). Thus, the field needs accessible biomarkers that distinguish RRMS from PMS early in MS course.^20^

Tau protein forms, well-known biomarkers of neurodegenerative diseases, may complement NfL and GFAP in MS.^21–25^ Previous studies reported tau accumulation in CSF of pwMS relative to non-inflammatory neurological controls.^26–28^ Abnormal tau phosphorylation was observed in brain tissue of pwMS and experimental autoimmune encephalomyelitis models, suggesting tau pathology as relevant in MS.^29–31^ Notably, tau phosphorylation in the CSF of PPMS was higher than RRMS.^32^ To the best of our knowledge, there has been no report of blood tau biomarkers for MS.

Here, we measured plasma total tau (t-tau), phosphorylated tau 181 (p-tau181), phosphorylated tau 217 (p-tau217), NfL and GFAP by leveraging the biobank of a clinic-based prospective MS cohort. We examined whether plasma tau proteins provide additional clinical utility beyond existing biomarkers (i.e. NfL and GFAP) in MS diagnosis, subtype differentiation, and prognosis (of long-term multi-modal outcomes).

## Materials and methods

### Ethics approval

The University of Pittsburgh Institutional Review Board approved the study protocols (STUDY19080007). All participants provided written informed consent.

### Study population

The study population were participants enrolled in the Prospective Investigation of Multiple Sclerosis in the Three Rivers Region (PROMOTE) between 2017 and 2023. PROMOTE is a clinic-based prospective cohort (UPMC, Pittsburgh) including pwMS and controls.^33–40^ For this study, we defined pwMS as having a neurologist-confirmed MS diagnosis according to the 2017 McDonald criteria.^41^ We defined controls as participants without a diagnosis of MS or related disorders (i.e. neuromyelitis optica spectrum disorder, myelin oligodendrocyte glycoprotein antibody-associated disease and other neuroimmunological disorders). Eligible participants had at least one plasma sample.

### Exposure: plasma biomarkers

Participants donated venous blood samples during routine clinic visits. Plasma samples were isolated and stored per standard protocol until assay. We selected the first available plasma sample of each participant as baseline, which might not be at diagnosis. We quantified baseline plasma levels of p-tau181, p-tau217, t-tau, NfL and GFAP using ultrasensitive single molecule array (Simoa) immunoassays. Detailed assay protocols, quality control procedures, and validation data are in Supplementary Methods 1.

### Outcomes

Beyond MS diagnosis and subtype, we examined multi-modal patient-reported and rater-assessed disability measures, functional status and imaging outcomes: e.g. Patient Determined Disease Steps (PDDS), age-related MS severity score (ARMSS) based on Expanded Disability Status Scale (EDSS), timed 25-foot walk (T25-FW), nine-hole peg test (9-HPT) and Symbol Digit Modalities Test (SDMT), retinal nerve fibre layer (RNFL) thickness, and total and grey matter brain volumes. Outcome definitions and procedures are in Supplementary Methods 2. We excluded participants with missing outcomes over the follow-up in prognosis analyses.

### Covariates

We collected demographic and clinical data from cohort registry and confirmed via review of electronic health records: age (at blood sample collection), sex, self-reported race and ethnicity (non-Hispanic white versus otherwise), height and weight, disease subtypes (RRMS, PPMS and SPMS), disease duration (years between MS diagnosis and sample collection), DMT effectiveness (none, standard and high), baseline PDDS (i.e. score reported on the day closest to sample collection) and relapse history 1 year before the sample collection. Obesity status was based on body mass index (BMI ≥ 30 kg/m2) as the waist size was unavailable. We combined PPMS and SPMS under PMS, given the modest sample size of each subtype. For DMT, we operationally categorized natalizumab, ocrelizumab, ofatumumab, rituximab and cladribine as high-effectiveness, while dimethyl fumarate, fingolimod, glatiramer acetate, interferon beta and teriflunomide as standard-effectiveness. All participants who had available baseline plasma biomarkers had complete information on covariates.

### Statistical analysis

Participant characteristics were presented as mean [standard deviation (SD)], median [IQR (interquartile range)] or frequencies and proportions, depending on the type and distribution of the data. We assessed correlations among biomarkers using Spearman correlation, and examined associations of biomarkers with sex, race/ethnicity and disease duration using Wilcoxon rank-sum tests or Kruskal–Wallis tests, as appropriate. We assessed the association of tau biomarkers with MS diagnosis (i.e. MS versus controls), adjusting for age and sex. We categorized pwMS into RRMS and PMS and tested the difference in tau biomarker values between MS subtypes and to controls. Dunn’s test with Benjamini–Hochberg (BH) corrected *P*-value was used for pairwise comparison when appropriate.

Among pwMS, we used weighted logistic regression to assess cross-sectional associations between plasma biomarkers and the odds of having PMS. We used balancing weights to account for differences in the distributions of age, sex, race and ethnicity and disease duration between PMS and RRMS. Specifically, all PMS participants were assigned a weight of 1, and RRMS participants were reweighted so that their covariate distributions matched those of PMS (details in Supplementary Methods 3). This created a pseudo-population in which demographic confounding was minimized. Compared with conventional multivariable adjustment, which relies on correctly specifying the model (including nonlinear forms and interactions), balancing weights directly standardize covariates, yielding more stable and interpretable estimates and reducing the risk of bias when association with covariates (e.g. relationship between tau markers and age and disease duration) may be nonlinear or not well understood. We then fit logistic regression model in the pseudo-population to estimate the odds of PMS per 1 SD increase in the biomarker value. We first entered each single biomarker separately (Model 1) to evaluate its individual association with PMS. We then entered all biomarkers (i.e. p-tau181, p-tau217, t-tau, NfL and GFAP) simultaneously (Model 2) to assess the independent associations of each biomarker with PMS while adjusting the other biomarkers. To compare the ability of tau biomarkers to distinguish PMS from RRMS with clinical features (age, sex, race and ethnicity, obesity, disease duration and DMT effectiveness) and benchmark biomarkers (NfL and GFAP), we used the area under the receiver operating characteristics curve (AUROC) and the decision curve analyses for models with different sets of predictors (i.e. Set 1: clinical features alone; Set 2: clinical features, NfL and GFAP; Set 3: clinical features and tau biomarkers; Set 4: all predictors together). AUROC analysis evaluated model accuracy while decision curve analysis assessed prediction utility regarding clinical value (Supplementary Methods 4). In addition, we estimated other standard metrics of model performance including accuracy, F1 score, sensitivity, specificity, positive predictive value and negative predictive value using 500 repetitions of 5-fold cross-validation. The approach mitigated overestimation of the model performance. Model performance metrics were averaged across repetitions to provide robust estimates. No stepwise regression was performed in these analyses. All models were pre-specified to evaluate the individual and independent associations of biomarkers with PMS and contributions to differentiate PMS from RRMS.

Among pwMS with ≥1 assessment of target outcomes taken ≥3 months after completing the baseline blood draw, we examined longitudinal associations between baseline biomarkers and subsequent multi-modal outcomes. We used generalized estimating equation (GEE) models to estimate the relationship between biomarkers with repeatedly measured PDDS, T25-FW, 9-HPT, SDMT, RNFL thickness, normalized total brain volume and normalized grey matter volume over the follow-up. All GEE models used an unstructured correlation matrix and a robust covariance matrix. For each outcome, we reported the overall and independent change per 1 SD increase in the biomarker concentration by entering each biomarker separately and then entering all biomarkers simultaneously. All models were adjusted for age, sex, race and ethnicity, disease duration, obesity status, MS subtype, baseline PDDS, DMT effectiveness and 1-year relapse history. Additionally, we conducted supplementary analyses using tertile-based models and sensitivity checks for outcomes assessed ≥6 months post-baseline. These details are reported in Supplementary Methods 5.

Statistical significance for the cross-sectional and longitudinal associations was defined as *P* < 0.05. We did not correct for multiple comparisons in these analyses because the goal was to compare tau biomarkers with benchmark GFAP and NfL. Statistical analyses were performed using R version 4.3.2.

## Results

### Cohort characteristics

**Figure 1:**
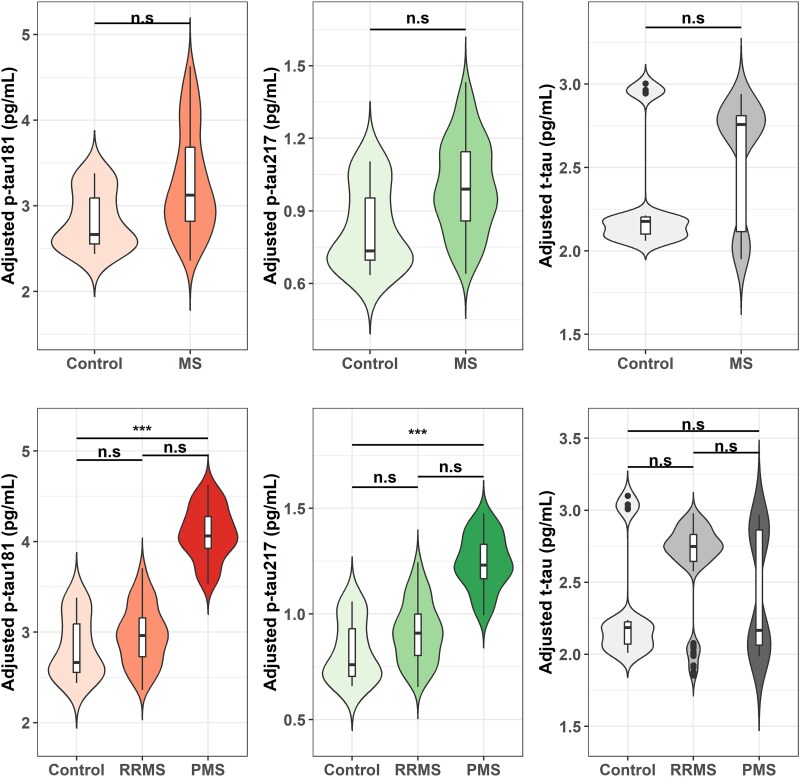
**Age- and sex-adjusted tau marker values by diagnosis groups (*N* = 180).**  *X*-axis represents the diagnosis groups. *Y*-axis represents age and sex- adjusted tau marker values using multivariable linear regression [controls = 20; MS = 160 (RRMS = 117, PMS = 43)]. Statistical comparisons were performed using Wilcoxon rank-sum tests for MS versus controls and Dunn’s test with BH correction for pairwise comparisons among controls, RRMS and PMS. RRMS, relapsing–remitting MS; PMS, progressive MS; p-tau181, phosphorylated tau 181; p-tau217, phosphorylated tau 217; t-tau, total tau. Significance thresholds: ***P < 0.001; **P < 0.01; *P < 0.05; ^ns^*P* > 0.05.

In the clinic-based cohort of 180 participants (pwMS: *n* = 160, 88.9%; controls: *n* = 20, 11.1%) with baseline plasma samples, the predominantly non-Hispanic White (*n* = 164; 91.1%) and women (*n* = 123, 68.3%) demographics were representative of the broader clinic population (Table 1). One hundred and seventeen (73.1%) pwMS had RRMS subtype at sample collection. Compared to controls, pwMS had significantly higher levels of plasma p-tau217 (median = 1.0 versus 0.7 pg/ml, *P* = 0.04) and NfL (median = 14.1 versus 9.0 pg/ml, *P* < 0.01), while other biomarkers had limited diagnostic value in distinguishing pwMS from controls. Figure 1 indicated age and sex-adjusted tau markers across disease diagnosis groups. Tau levels (i.e. p-tau181, p-tau217 and t-tau) were statistically similar between pwMS and controls, while PMS had a significantly higher p-tau181 (adjusted mean: 4.1 versus 2.8 pg/ml; BH corrected *P* < 0.01) and p-tau217 (adjusted mean: 1.2 versus 0.8 pg/ml; BH corrected *P* < 0.01) than controls.

**Table 1:** Participant characteristics

	Overall	MS	Control	*P*-value^a^
*n*	180	160	20	
Age, years; median (IQR)	51.20 (40.11, 61.18)	52.75 (41.16, 61.18)	42.50 (33.45, 55.17)	0.07
Sex; *n* (%)				<0.01
Women	123 (68.3%)	119 (74.4%)	4 (20.0%)	
Men	57 (31.7%)	41 (25.6%)	16 (80.0%)	
Race and ethnicity; *n* (%)				0.22
Non-Hispanic White	164 (91.1%)	144 (90%)	20 (100%)	
Other	16 (8.9%)	16 (10%)	0 (0%)	
Obesity; *n* (%)	60 (33.3%)	53 (33.1%)	7 (35.0%)	0.55
MS subtype; *n* (%)				N/A
RRMS	N/A	117 (73.1%)	N/A	
PPMS	N/A	24 (15.0%)	N/A	
SPMS	N/A	19 (11.9%)	N/A	
MS duration, years; median (IQR)	N/A	12.10 (3.79, 19.46)	N/A	N/A
DMT effectiveness^b^				N/A
None	N/A	40 (25.0%)	N/A	
Standard	N/A	51 (31.9%)	N/A	
High	N/A	69 (43.1%)	N/A	
p-tau181, pg/ml; median (IQR)	3.28 (2.24, 4.22)	3.38 (2.22, 4.27)	2.88 (2.27, 3.29)	0.11
p-tau217, pg/ml; median (IQR)	0.97 (0.68, 1.34)	1.02 (0.69, 1.38)	0.74 (0.66, 0.93)	0.04
t-tau, pg/ml; median (IQR)	2.76 (1.78, 3.78)	2.80 (1.79, 3.81)	2.59 (1.71, 3.38)	0.32
NfL, pg/ml; median (IQR)	13.56 (8.91, 19.55)	14.07 (9.36, 20.13)	8.97 (7.53, 12.71)	<0.01
GFAP, pg/ml; median (IQR)	47.82 (26.04, 82.98)	49.82 (26.05, 86.60)	36.56 (24.82, 63.47)	0.11

p-tau181, phosphorylated tau 181; p-tau217, phosphorylated tau 217; t-tau, total tau; GFAP, glial fibrillary acidic protein; NfL, neurofilament light chain; RRMS, relapsing–remitting MS; PPMS, primary progressive MS; SPMS, secondary progressive MS; DMT, disease-modifying therapy. ^a^*P*-value of the Wilcoxon rank-sum test for continuous measures and the Fisher’s exact test for categorical measures. ^b^High-effectiveness DMT included natalizumab, ocrelizumab, rituximab, cladribine and ofatumumab; Standard-effectiveness DMT included dimethyl fumarate, fingolimod, glatiramer acetate, interferon beta and teriflunomide.

### Characteristics of tau biomarkers in pwMS

There were significant positive correlations between p-tau181 and p-tau217 (corr = 0.69, *P* < 0.01), and p-tau181 and t-tau (corr = 0.27, *P* < 0.01) (Supplementary Fig. 1A). NfL correlated with GFAP (corr = 0.39, *P* < 0.01) and were both positively correlated with p-tau217 (Supplementary Fig. 1A). We observed a nonlinear relation with age for all biomarkers (Supplementary Fig. 1B). T-tau was significantly higher in females than in males (median concentration: 3.13 versus 2.16 pg/ml; *P* < 0.01; Supplementary Fig. 1C). Otherwise, no significant difference was observed for subgroups of sex and race/ethnicity (Supplementary Fig. 1C and D). All biomarkers except t-tau showed statistically significant differences across disease duration groups (Supplementary Fig. 1E).

### Progressive MS and plasma tau biomarkers

**Figure 2:**
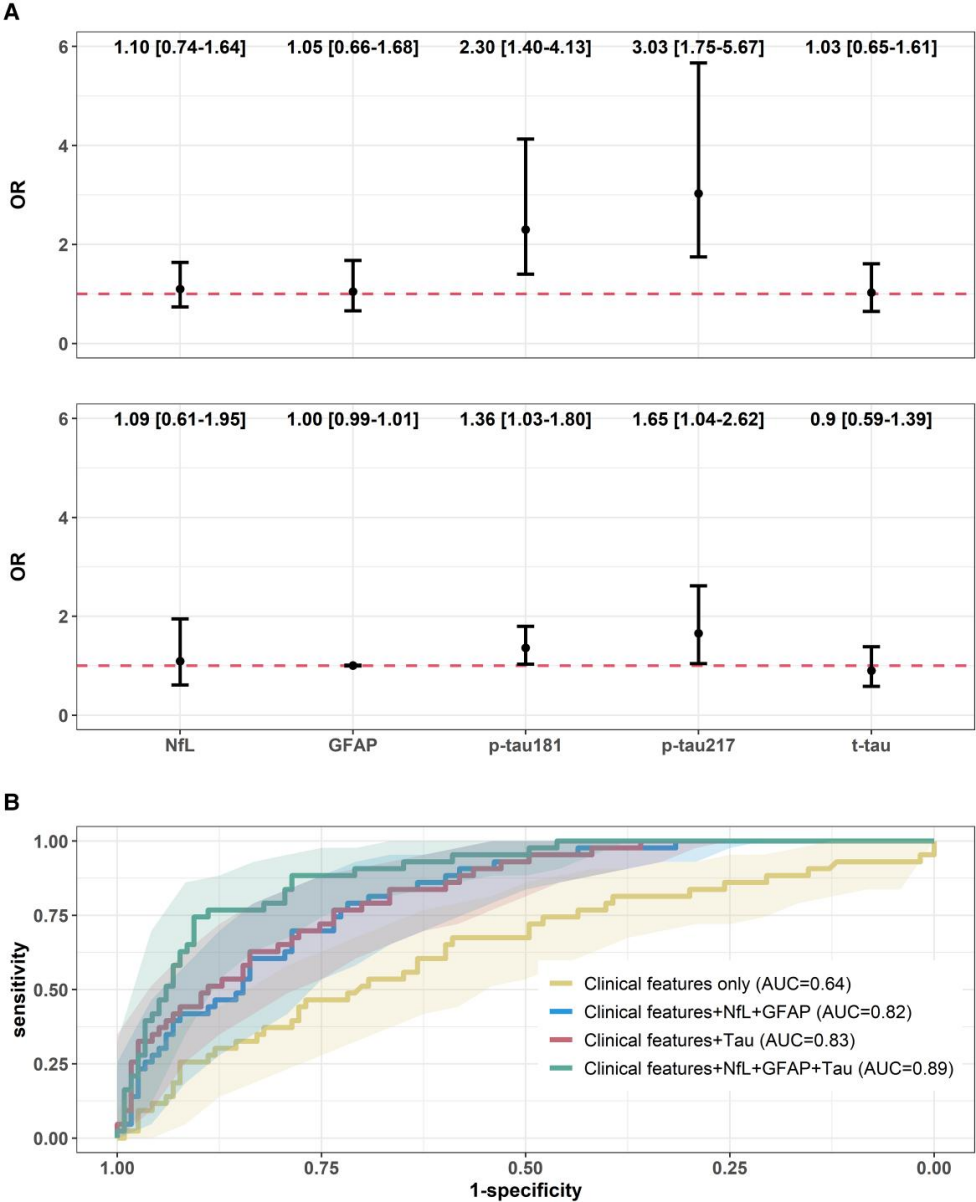
**Biomarkers and MS subtypes (*N* = 160).** (**A**) Blood biomarker concentration and odds of PMS. The results were derived from weighted logistic regression models with balancing weights on age, sex, race and ethnicity and disease duration. Estimates are OR and corresponding 95% CI for a 1 SD increase in each biomarker concentration. The horizontal dashed line represents a null association of OR = 1. In Model 1, separate logistic regression models were run for each biomarker. In Model 2, all markers were entered simultaneously to assess the adjusted association independent of other biomarkers. (**B**) ROC and corresponding 95% CI of different sets of predictors for distinguishing MS subtypes. The value of AUC indicates model performance in distinguishing PMS from relapse-remitting MS. Fifty percentage indicates no difference from chance and 100% indicates a perfect ability of distinction with sensitivity and specificity of 100%. Clinical features included age, sex, race and ethnicity, obesity status, disease duration and DMT effectiveness. Tau included p-tau181, p-tau217 and t-tau. p-tau181, phosphorylated tau 181; p-tau217, phosphorylated tau 217; t-tau, total tau; GFAP, glial fibrillary acidic protein; NfL, neurofilament light chain; OR, odds ratio.

Among the 160 pwMS, we applied the balancing weights method to assess the association between biomarker concentrations and the odds of having PMS. The distribution of age, sex, race and ethnicity and disease duration were comparable between PMS and RRMS after balancing weights (Table 2). In the weighted cohort (i.e. pseudo-population after weighting RRMS to PMS demographics), elevated p-tau181 {odds ratio (OR) [95% confidence interval (CI)] = 2.30 [1.40, 4.13]} and p-tau217 [OR (95% CI) = 3.03 (1.75, 5.67)] were each associated with higher odds of PMS, whereas t-tau, NfL and GFAP were not (Fig. 2A). After adjusting for other biomarkers, higher p-tau181 [OR (95% CI) = 1.36 (1.03, 1.80)] and p-tau217 [OR (95% CI) = 1.65 (1.04, 2.62)] maintained associations with higher odds of PMS (Fig. 2A).

**Table 2:** Characteristics of progressive MS versus relapsing–remitting MS before and after applying balancing weights

	Progressive MS	Relapsing–remitting MS	Relapse-remitting MS with balancing weights
*n*	43	117	117
Age, years; median (IQR)	61.12 (56.66, 67.86)	48.06 (36.06, 55.82)	63.84 (57.08, 72.38)
Age category; *n* (%)			
≤40 years	4 (7.1%)	47 (40.2%)	10.2 (8.7%)
40–60 years	15 (35.7%)	49 (41.9%)	37.1 (31.8%)
>60 years	24 (57.1%)	21 (18.0%)	69.6 (59.5%)
Sex; *n* (%)			
Women	21 (47.6%)	98 (84.6%)	64.1 (54.8%)
Men	22 (52.4%)	19 (15.4%)	52.9 (45.2%)
Race and ethnicity; *n* (%)			
Non-Hispanic White	36 (85.7%)	108 (91.5%)	100.5 (85.9%)
Other	7 (14.3%)	9 (8.5%)	16.5 (14.1%)
Disease duration, years; median (IQR)	15.85 (6.58, 24.91)	10.43 (3.49, 17.55)	17.55 (10.88, 22.98)
Disease duration category; *n* (%)			
≤5 years	11 (23.8%)	38 (32.5%)	22.7 (19.4%)
5–15 years	9 (21.4%)	40 (34.2%)	21.3 (18.2%)
>15 years	23 (54.7%)	39 (33.3%)	73.0 (62.4%)

As predictors, Tau biomarkers (p-tau181, p-tau217 and t-tau) along with clinical features, GFAP and NfL distinguished PMS from RRMS with clinically actionable accuracy [area under the curve (AUC) (95% CI) = 0.89 (0.84, 0.94); Fig. 2B; Supplementary Table 1]. This combined feature set outperformed clinical features alone [AUC = 0.64 (0.54, 0.74)], clinical features with GFAP and NfL [AUC = 0.82 (0.75, 0.86)] and clinical features with tau biomarkers [AUC = 0.83 (0.76, 0.89)]. Decision curve analysis quantified the clinical utility of models by net benefit. Adding tau biomarkers as predictors to clinical features, NfL and GFAP yielded increased net benefit across a wide range of threshold probability (Supplementary Fig. 2). Considering pwMS with a predicted PMS risk of 0.5 (i.e. at 50% probability threshold, 50% chance to be PMS versus RRMS), incorporation of tau biomarkers added a ∼5% higher net benefit than the prediction model containing clinical features, GFAP and NFL (Supplementary Table 1). Other metrics of MS subtype classification models were shown in Supplementary Table 2.

### Multi-modal outcomes and plasma tau markers

**Figure 3:**
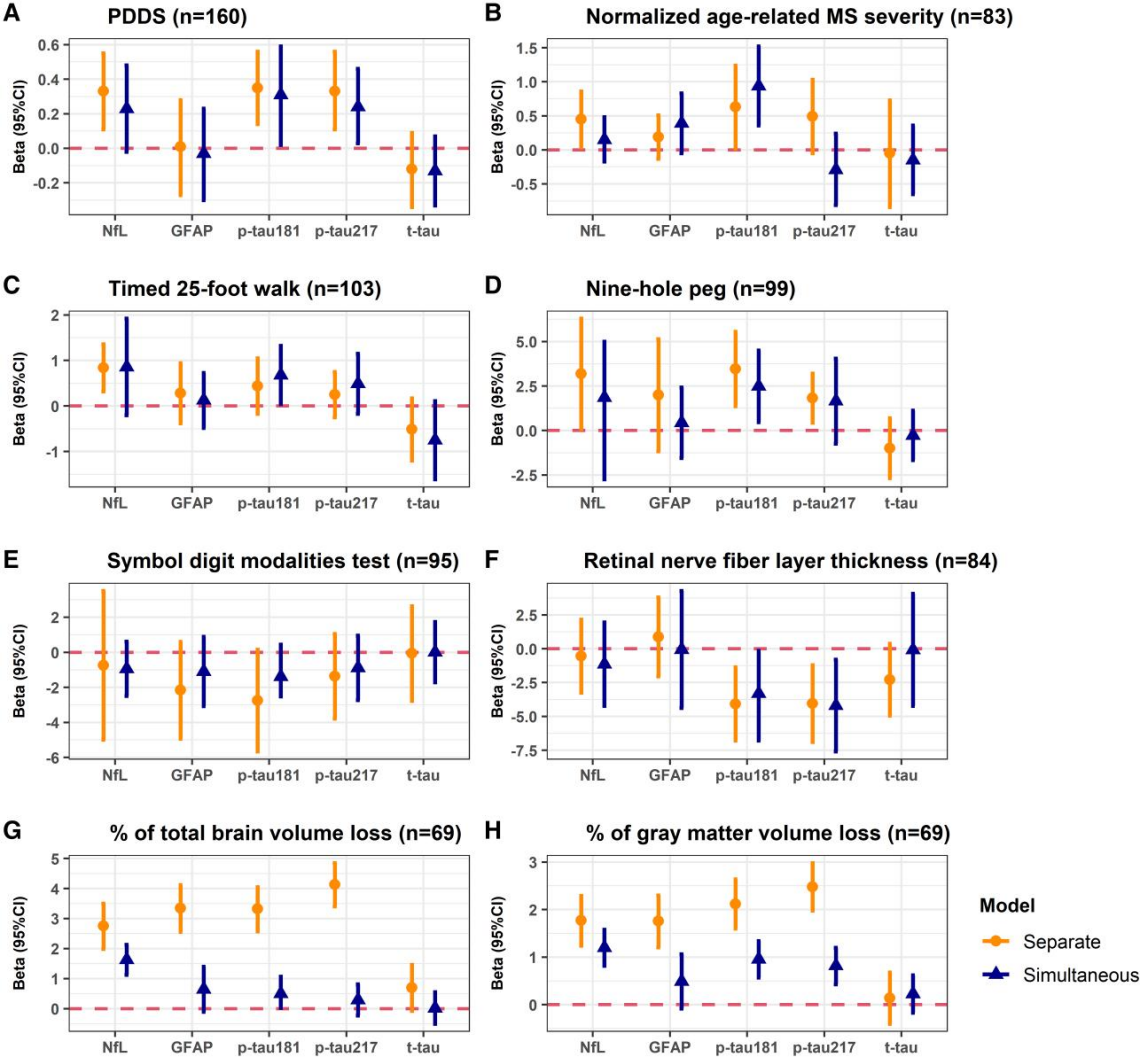
**Associations between baseline biomarker concentration and multi-modal outcomes 3 months after baseline using generalized equation estimation models.** (**A**) PDDS) *n* = 160. (**B**) Normalized ARMSS; *n* = 83. (**C**) T25-FW; *n* = 103. (**D**) 9-HPT (*n* = 160); *n* = 99. (**E**) SDMT correct score; *n* = 95. (**F**) RNFL thickness; *n* = 84. (**G**) Per cent total brain volume loss; *n* = 69. (**H**) Percent grey matter volume loss; *n* = 69. Generalized equation estimation models were used to estimate associations between biomarkers and repeatedly measured outcomes during follow-up. Models were adjusted for age, sex, race and ethnicity, disease duration, obesity status, MS subtype, baseline PDDS, DMT effectiveness and 1-year relapse history. Estimates and corresponding 95% CI for a 1 SD increase in each marker concentration are displayed. The horizontal dashed line represents a null association of beta = 0. In ‘separate’ models, each biomarker was separately entered. The estimates are the change in the outcome per 1 SD increase in the biomarker value. In the ‘simultaneous’ models, all biomarkers were simultaneously entered. The estimates are the change in the outcome per 1 SD increase in the biomarker value independent of other biomarkers. p-tau181, phosphorylated tau 181; p-tau217, phosphorylated tau 217; t-tau, total tau; GFAP, glial fibrillary acidic protein; NfL, neurofilament light chain.

The median (IQR) of the cohort follow-up was 3.0 (1.2, 4.7) years. Figure 3 and Supplementary Table 3 presented associations and dose–response trends between baseline biomarkers and longitudinal multi-modal outcomes 3 months post-baseline (i.e. after sample collection), adjusting for age, sex, race and ethnicity, disease duration, obesity status, MS subtype, baseline PDDS, DMT effectiveness and 1-year relapse history. Results on outcomes 6 months post-baseline were reported in Supplementary Results, Supplementary Table 4 and Supplementary Fig. 3. Separate models captured each marker’s total association with outcomes, whereas simultaneous models estimated the independent contribution of each marker after accounting for others. Accordingly, some associations attenuate or lose significance when shared variance is partitioned in simultaneous models. Notably, when a biomarker remained significant in the simultaneous models, it indicated additive predictive value for the outcome beyond that explained by other markers.

### Disability: patient-reported (PDDS) and rater-assessed (normalized age-related MS severity)

All pwMS (*n* = 160) had ≥1 observations of PDDS 3 months post-baseline. Elevated p-tau181, p-tau217 and NfL were each associated with worse PDDS score (beta range: 0.33–0.35; Fig. 3A), and all three biomarkers reached significant dose–response trends when modelling biomarkers as tertiles (Supplementary Table 3). Estimates were the change in the outcome per 1 SD increase in the biomarker concentration. The association remained significant for p-tau181 [beta (95% CI) = 0.31 (0.02, 0.59)] and p-tau217 [beta (95% CI) = 0.24 (0.03, 0.46)], after adjusting for other biomarkers.

Eighty-three pwMS had ≥2 observations of EDDS 3 months post-baseline to compute normalized ARMSS. Elevated p-tau181 concentration was associated with worse ARMSS, when modelling separately [beta (95% CI) = 0.63 (0.02, 1.24)] and simultaneously [beta (95% CI) = 0.94 (0.36, 1.52)] (Fig. 3B). We did not observe any significant trend between any plasma biomarker and normalized ARMSS (Supplementary Table 3).

### Functional outcomes: walk speed (T25-FW), manual dexterity (9-HPT), and cognition (SDMT)

When modelling each biomarker individually to predict rater-assessed functional status, elevated p-tau181, p-tau217 and NfL concentrations were associated with worse manual dexterity (higher 9-HPT), while only elevated NfL was associated with slower walk speed (higher T25-FW) (Fig. 3C and D). When modelling all biomarkers simultaneously, 1 SD elevation of p-tau181 was independently associated with worse walk speed [beta (95% CI) = 0.68 (0.04, 1.32)] and worse manual dexterity [beta (95% CI) = 2.49 (0.47, 4.50)], whereas no other markers showed significant association. p-tau181 and NfL showed dose–response trends with manual dexterity (Supplementary Table 3). While both p-tau181 and p-tau217 showed dose–response trends with cognition (lower correct SDMT score) when separating concentrations into tertiles in a data-driven manner, there was no significant association between continuous biomarker concentrations and cognition (Fig. 3E; Supplementary Table 3). Relative to pwMS in the bottom tertile, pwMS in the top tertile of p-tau181 and p-tau217 had 3.15 and 2.41 fewer numbers of SDMT correct scores, respectively.

### Optical coherence tomography (OCT) outcome: RNFL thickness

One SD increase in the concentration of p-tau181 and p-tau217 predicted a 4.08 µm [(95% CI) = (1.37, 6.78)] and 4.05 µm [95% CI = (1.21, 6.89)] decrease of RNFL thickness during follow-up, respectively (Fig. 3F). Further, higher baseline p-tau181 [beta (95% CI)=–3.31 (−6.78, −0.16)] and p-tau217 [beta (95% CI) = −4.20 (−7.59, −0.81)] were associated with lower RNFL thickness, independent of other biomarkers, whereas t-tau, NfL and GFAP were not. A significant trend was detected for p-tau217, as pwMS in the top tertile of p-tau217 had a mean of 7.01 µm lower in RNFL thickness relative those in the bottom tertile (Supplementary Table 3).

### MRI outcome: total brain volume and grey matter volume normalized to the intracranial volume

All biomarkers except for t-tau were separately associated with normalized total brain volume (TBV) and grey matter value (GMV; beta range: 2.75–4.13% for TBV, 1.76–2.48% for GMV; Fig. 3G and H). After adjusting for other biomarkers, elevated p-tau181 [beta (95% CI) = 0.50 (0.03, 1.07)] and NfL [beta (95% CI) = 1.63 (1.13, 2.13)] were independently associated with greater TBV loss, while elevated p-tau181 [beta (95% CI) = 0.96 (0.57, 1.34)], p-tau217 [beta (95% CI) = 0.82 (0.43, 1.20)] and NfL [beta (95% CI) = 1.20 (0.82, 1.58)] were independently associated with greater GMV loss. p-tau217 and NfL each showed a significant dose–response trend for total brain and grey matter atrophy (Supplementary Table 3), while p-tau181, t-tau and GFAP did not.

## Discussion

In this clinic cohort study, plasma tau biomarkers showed limited diagnostic utility, but potential subtyping and prognosis clinical utility in MS. Tau biomarkers did not differentiate pwMS from controls. Higher p-tau181 and p-tau217 levels were associated with increased odds of PMS (versus RRMS), improving subtyping distinction beyond clinical features, NfL and GFAP. Importantly, plasma p-tau181 and p-tau217 showed prognostic potential as they contributed additional value beyond NfL and GFAP in predicting multi-modal disability, functional and neuroimaging outcomes.

To the best of our knowledge, this is the first study to comprehensively investigate the clinical potential of *blood*-based tau proteins as MS biomarkers. As an axonal cytoskeletal protein, tau is essential for CNS health and an established biomarker of neurodegeneration (e.g. Alzheimer’s disease).^42,43^ Axonal damage and neuronal loss began in early MS stages.^44,45^ Neuropathological and pre-clinical studies reported abnormally phosphorylated tau in MS that may form neurotoxic tau aggregates.^29,30^ Emerging studies evaluated CSF tau, while few studied blood tau in pwMS.^46^ While detectable, blood tau concentrations were estimated to be 10 times lower than CSF.^47^ Recent advances in ultrasensitive immunoassays enabled reliable detection of blood-based tau, offering a less invasive and more accessible alternative for biomarker studies. Leveraging ultrasensitive immunoassays, rigorously annotated patient characteristics and longitudinal multi-modal outcomes, we assessed the associations of plasma tau markers with MS diagnosis, subtype and prognosis. We chose to analyse plasma rather than serum primarily because of the compelling evidence of high inter-assay correlations across academic research laboratories and biotechnology / pharmaceutical companies when assaying plasma p-tau isoforms (p-tau181, p-tau217 and p-tau231).^48^ This would support the generalizability of our findings to other Simoa assays. Given the strong correlations between paired serum and plasma p-tau181 levels, we could evaluate the clinical utility of serum p-tau181 and p-tau217 in the future.^49,50^

First, we reported limited diagnostic value for plasma tau biomarkers. Adjusting for age and sex, tau levels (i.e. t-tau, p-tau181 and p-tau217) in pwMS and controls did not differ significantly. Indeed, the literature of tau and p-tau as diagnostic biomarkers in MS has produced contradictory results. Several factors may explain the discrepancy: (i) propensity of tau proteins to degradation with improper sample handling; (ii) substantial heterogeneity in MS populations; and (iii) differences in biospecimen type (CSF versus blood), assay methods, sample sizes and covariate adjustments.^25,51^ Some studies reported higher tau or p-tau levels in MS compared with controls, whereas others found no difference. For example, multiple reports found CSF t-tau levels in MS comparable to controls,^52–54^ while others noted increased CSF t-tau.^26,27,55^ In blood, one study reported lower serum t-tau concentrations in female pwMS compared with healthy controls, though this result may have been confounded by the lack of adjustment for age and disease heterogeneity (e.g. duration, subtype, DMT).^56^ Regarding CSF p-tau 181, Bartosik-Psujek and Stelmasiak^28^ observed elevated level in MS, whereas Masiulienė *et al*.^57^ reported undetectable level in early-stage MS and Emeršič *et al*.^58^ found no difference between MS and controls. For CSF p-tau217, one study reported levels in controls were significantly higher than in clinical isolated syndrome (CIS) but comparable to RRMS and PMS.^57^ Overall, these inconsistent findings underscore that tau biomarkers have context-dependent diagnostic utility in MS, highlighting the need for standardized assays and well-characterized cohorts in future validations.

After categorizing pwMS into PMS and RRMS here, PMS (but not RRMS) showed significantly higher age- and sex-adjusted levels of p-tau181 and p-tau217 than controls. The observation in our study that tau biomarkers did not significantly differentiate pwMS from controls whereas p-tau181 and p-tau217 did distinguish PMS from RRMS possibly reflects the biology of tau phosphorylation, which is more likely to inform chronic neurodegenerative processes than inflammatory demyelination. As such, RRMS patients, particularly those in earlier disease course, may show blood tau levels closer to controls, limiting the case-control distinction. In contrast, the chronic neurodegeneration and axonal loss in PMS are more directly tied to abnormal tau phosphorylation, potentially explaining the clearer differences between PMS and RRMS. This explanation is consistent with prior CSF studies showing higher phosphorylated tau in PMS than RRMS, but not consistently higher in overall MS versus non-MS controls.^29,30^

Next, we showed the subtyping utility of plasma p-tau biomarkers to differentiate PMS from RRMS. Neurodegeneration largely drives the RRMS transition to SPMS and the worsening disability in PMS.^1,2,59^ As one potential explanation for the observed association between p-tau and PMS, immune cell activation promotes oxidative stress, and subsequent axonal tau phosphorylation and degeneration.^60^ Serum NfL and GFAP could stratify pwMS into different progression and disease activity status.^61^ We observed a similar finding for plasma NfL and GFAP, but crucially highlighted the additive value of plasma p-tau181 and p-tau217 in MS subtyping in combination with NfL and GFAP. The synergistic approach could improve MS subtype classification and inform timely treatment strategies given the expected growing DMT options for PMS.

Finally, our study suggested the potential prognostic utility of plasma p-tau biomarkers. p-tau181 and p-tau217 (but not t-tau) consistently added predictive value for post-baseline multi-modal outcomes beyond NfL and GFAP. Prior CSF t-tau studies were inconclusive regarding association with MS disability. Martínez-Yélamos *et al.*^62^ and Virgilio *et al.*^63^ reported CSF t-tau predictive of poor outcomes in early RRMS, but Gajofatto *et al.*^64^ found CSF t-tau not predictive of disability after ∼6 years of follow-up in pwMS. No study has investigated plasma p-tau and MS progression. We showed consistent p-tau associations with multi-modal patient-reported and rate-assessed clinical outcomes and neuroimaging outcomes. Consistent associations with validated clinical outcomes of disability (i.e. PDDS, normalized ARMSS derived from EDSS) and objective measures of neurodegeneration (e.g. functional performance, RNFL thickness, and brain atrophy) strongly support their prognostic utility.

An interesting study observation was that the raw and age-adjusted distributions of t-tau were unimodal, whereas the sex-adjusted distribution displayed bimodality. This finding suggests that sex is associated with systematic differences in t-tau levels, and adjusting for sex may reveal underlying subgroup structure rather than producing a single centred distribution. This explanation not only was supported by the finding of significantly higher t-tau concentrations in females than males but also was consistent with previous reports of sex differences in tau biology and pathology. Prior studies in Alzheimer’s disease and Down syndrome reported greater tau accumulation and vulnerability to tau-mediated neurodegeneration in women.^65–69^ Our findings highlight the importance of considering sex-specific variation when interpreting tau biomarker data. Future studies of the biological mechanisms (e.g. sex hormones) underlying these differences are necessary.^70^

No prior study has concurrently assessed blood-based t-tau and p-tau in MS. Our observation that p-tau181 and p-tau217, but not t-tau, showed stronger and more consistent associations with MS subtype and prognostic outcomes is consistent with insights from Alzheimer’s disease research. Plasma t-tau has shown limited diagnostic or prognostic value because it reflects general axonal injury and may include contributions from peripheral sources, resulting in poor disease specificity and inconsistent associations across studies.^48,68^ Indeed, the weak correlation between blood and CSF t-tau suggests that only a minority of plasma t-tau (∼20%) is CNS-derived, making it difficult to capture CNS neurodegeneration.^25^ In contrast, p-tau biomarkers (e.g. p-tau181 and p-tau217) are thought to reflect the same pool of brain-secreted p-tau as in CSF.^71^ These biomarkers have consistently demonstrated superior performance, showing strong correlation with CSF p-tau and tau PET imaging, tracking with disease stage and predicting cognitive decline and clinical progression more reliably than t-tau. Beyond Alzheimer’s disease, a study comparing amyotrophic lateral sclerosis to controls similarly found group differences in serum p-tau, but not t-tau, further supporting the advantage of p-tau.^71,72^ Taken together, these findings suggest that tau phosphorylation captures pathophysiological processes directly linked to abnormal tau aggregation and neurodegeneration, whereas t-tau provides a more non-specific measure of neuronal injury. Beyond p-tau181 and p-tau217, other phosphorylated tau epitopes such as p-tau212, p-tau231, p-tau262 and p-tau356 may also play complementary roles at different disease stages.^58,73,74^ Although not included in the present study, these represent promising targets for future biomarker investigations in MS.

Our study has several strengths. First, it has the largest sample size to date for investigating fluid (e.g. blood and CSF) tau proteins in pwMS. Second, assays of all biomarkers using the same high-sensitivity platform and standardized protocol enhance the reliability and reproducibility of our exposure assessments. Third, we evaluated the clinical potential of tau biomarkers against the current benchmark MS biomarkers (i.e. GFAP and NfL) and quantified the added clinical value of tau markers alongside NfL and GFAP. Fourth, we comprehensively examined plasma tau as biomarkers of MS diagnosis, subtype differentiation and prognosis using rigorous statistical strategies and minimizing confounding biases. Finally, our clinic-based cohort study incorporated multi-modal longitudinal outcomes collected during routine clinic care, including metrics from clinical OCT and MRI. These clinically available multi-modal outcomes enabled evaluation of plasma tau biomarkers in a real-world setting.

Our study has limitations. The absence of external validation and relatively low proportion of racial and ethnic minorities restricted the broader generalizability of the findings. The modest sample size of controls precluded a formal receiver operating curve (ROC) analysis for MS diagnosis. Thus, the finding of the limited diagnostic utility of plasma tau biomarkers would need confirmation in future studies with larger control samples. Further, the pragmatic decision combining PPMS and SPMS as PMS given the sample size limited the biomarker evaluation in these two subtypes, which differ in pathological mechanisms. Finally, the median follow-up of 3.0 years was relatively short, constraining the evaluation of long-term prognostic utility of tau biomarkers. As the clinic-cohort study is ongoing, continued accrual of multi-modal clinical outcomes will enable reassessment of p-tau181 and p-tau217 over longer horizons. Future replications in independent cohorts with broader demographic representations, more controls and PMS individuals and longer follow-up are crucial. Nevertheless, our study serves as a foundational framework for future investigations of tau markers and tauopathies in MS.

## Conclusion

Plasma tau biomarkers, particularly p-tau181 and p-tau217, are potential biomarkers for MS subtype differentiation and outcome prediction. This study contributes towards the growing research on accessible biomarkers that could supplement existing tools to guide individualized management of pwMS. Beyond further validation of baseline blood tau biomarkers for clinical application, investigation of longitudinal tau biomarkers may provide mechanistic insights into MS progression.

## Supplementary Material

fcaf510_Supplementary_Data

## Data Availability

Code for analyses and figures is available at https://github.com/xialab2016/tau_biomarker. De-identified data are available upon request to the corresponding author and with permission from the participating institutions.
