# Supplementary material for: Plasmaphosphorylated tau as biomarkers for multiple sclerosis diagnosis, subtyping, and prognosis
Source: Brain Commun. 2026 Jan 2;8(1):fcaf510. doi: 10.1093/braincomms/fcaf510 (PMC12880184; doi:10.1093/braincomms/fcaf510)
Supplement: fcaf510_Supplementary_Data [file fcaf510_supplementary_data.pdf]

SUPPLEMENTARY MATERIAL

Supplementary Methods

Supplementary Methods 1. Plasma collection, Biomarker assays and quality control

Plasma samples from venous whole blood were isolated within four hours of phlebotomy following standard guidelines, stored at -80°C, and thawed at room temperature before biomarker assay. We centrifuged samples at 4000 x g for 10 minutes to remove particulates. We quantified plasma biomarkers using validated commercially available single molecule array (Simoa) assays from Quanterix on an HD-X instrument (Quanterix, Billerica, MA, USA). NfL and GFAP were measured using the Neurology 2-Plex assay (#103520). P-tau181, p-tau217, and t-tau were measured with the P-tau181 V2 Advantage kit (#103714), the ALZpath Simoa® P-Tau 217 V2 Assay Kit (#104371), and the Tau Advantage Kit (#101552), respectively. We assessed within-run and between-run precision by the coefficient of variation (CV). For each assay, we analyzed samples in three runs. Quality control samples of three different concentrations were analyzed at the beginning and end of each run. The average within-run CVs were 10.6% for NfL, 7.1% for GFAP, 6.1% for p-tau217, 10.9% for p-tau181, and 5.7% for t-tau. The average between-run CVs were 10.6% for NfL, 7.1% for GFAP, 17.3% for p-tau217, 12.6% for p-tau181, and 7.1% for t-tau. We used the average concentration of three runs as the biomarker level.

Supplementary Methods 2. Outcome assessment procedures and definitions

For assessing disability, we used a patient-reported outcome based on the PDDS scale and the normalized age-related MS severity score (ARMSS) derived from rater-assessed EDSS observations at ≥2 different time points. Normalized ARMSS is an age-ranked reliable metric that enables longitudinal comparison of disease course among pwMS.[1] For assessing functional tests, we quantified walking speed, manual dexterity, and cognitive function regularly by the timed 25-foot walk (T25-FW), nine-hole peg (9-HPT), and the symbol digit modalities test (SDMT), respectively. For imaging outcomes, we used

clinical data from optical coherence tomography (OCT, ZEISS Cirrus) and 3 tesla brain magnetic resonance imaging (MRI, GE) with NeuroQuant (Cortechs.ai), following clinical quality assurance. In this study, we focused on RNFL thickness, total brain volume, and gray matter volume, which are well-established neuroimaging markers for CNS neurodegeneration.[2,3] The multi-modal outcomes of this study include: (1) PDDS score; (2) normalized ARMSS; (3) T25-FW: the average of the two completed trials measuring in seconds; (4) 9-HPT: the average of the two completed trials for the dominant hand measuring in seconds; (5) SDMT: the score for the number of correct responses in 90 seconds; (6) RNFL thickness of both eyes measuring in μm; and (7) Total brain and gray matter volume normalized to the intracranial volume.

Supplementary Methods 3. Balancing weights for confounders adjustment

We used balancing weights, a statistical approach similar to inverse propensity score weighting, to adjust RRMS individuals to be similar to PMS individuals with respect to demographic factors and achieve causal inference with minimum confounding bias. The balancing weights approach outperforms inverse probability weights with more accurate and stable estimates when the overlap between the exposed and unexposed groups is poor.[4,5] Our data suggested a great demographic discrepancy between RRMS and PMS. For example, the median [IQR] age of RRMS was 48.1 [36.1, 55.8] years, while 61.1 [56.7, 67.9] years for PMS. Additionally, we considered the balancing weights approach preferable to conventional multivariable logistic regression approach in our case. Multivariable models adjust for covariates parametrically, which requires correct specification of non-linear forms and interactions. Because the relationships between tau marker levels and patient features (*e.g.*, age and disease duration) are not fully understood and may be non-linear, such models are at risk of misspecification, potentially introducing bias. In contrast, balancing weights directly equalize the distribution of covariates between PMS and RRMS participants before outcome modeling. This reduces dependence on parametric assumptions and aligns the estimand with our clinical question: the odds of PMS versus RRMS in a pseudo-population where RRMS participants resemble PMS participants with

respect to demographic and clinical features. In practice, we divided participants into 36 strata based on age category (18-40, 40-60, and 60+ years), sex (male and female), race and ethnicity (non-Hispanic white and otherwise), and disease duration (0-5, 5-15, and 15+ years). All PMS patients were assigned a weight of 1, whereas weights for RRMS patients were calculated as the number of PMS individuals in a particular stratum divided by the number of RRMS individuals. After the application of balancing weights, the distribution of age, sex, race and ethnicity, and disease duration were similar in RRMS and PMS patients (**Supplementary Table 1**). Balancing weights were used in logistic regression models to estimate the odds ratios of PMS as compared to RRMS with a 1 SD increase in the biomarker concentration. Second-order polynomial terms for age and disease duration were used to account for residual confounding from the stratification in generating weights.

Supplementary Methods 4. Decision curve analysis

Introduced by Vickers and Elkin in 2006, decision curve analysis is a technique to assess the effectiveness of prediction models and diagnostic tests.[6] This method aims to address the shortcomings of conventional statistical measures like discrimination and calibration, which do not offer direct insights into the clinical usefulness of these tools. Unlike receiver operator characteristic (ROC) analysis, which typically assesses the model accuracy, decision curve analysis assesses the utility of prediction models through the concept of overall net benefit. It's not uncommon that an accurate model has poor clinical value in terms of net benefit.[7] In brief, net benefit is calculated across a range of threshold probabilities, defined as the minimum probability of disease/condition at which further intervention would be warranted. Net benefit = sensitivity × prevalence – (1 – specificity) × (1 – prevalence) × w, where w is the odds at the threshold probability. In this use case, the value of the net benefit indicates the benefits of a true positive diagnosis (*e.g.,* identifying a true progressive multiple sclerosis [PMS] patient as PMS) minus the harms of a false positive diagnosis (diagnosing a true relapsing-remitting multiple sclerosis [RRMS] patient as PMS) for any threshold probability. We calculated net benefit over the threshold range (*i.e.*, 0-1) for four models with different sets of predictors (*i.e.*, set 1: clinical features alone; set 2: clinical features,

NfL and GFAP; set 3: clinical features and tau biomarkers; set 4: all predictors together). A higher net benefit suggests greater clinical utility of a subtype prediction model at a given threshold probability.

Supplementary Methods 5. Supplementary analysis for associations between markers and clinical outcomes

We alternatively modeled the biomarker concentration with categorical tertiles to accommodate dose-response relations, adjusting for the same covariates. Trend analysis was performed by including tertiles as a continuous covariate in models. Based on these models, we reported relative change in each outcome for 2nd and 3rd tertiles relative to the 1st tertile, and p-values for the dose-response trend. In sensitivity analyses, we evaluated whether associations of baseline markers and outcomes 6 months after baseline were consistent with that of 3 months after baseline by restricting included participants being pwMS who had ≥1 assessment of the outcome of interest taken ≥6 months after the baseline blood draw.

Reference

1. Manouchehrinia A, Kingwell E, Zhu F, Tremlett H, Hillert J, Ramanujam R. A multiple sclerosis disease progression measure based on cumulative disability. *Mult Scler*. 2021;27(12):1875-1883. doi:10.1177/1352458520988632

2. Saidha S, Sotirchos ES, Oh J, et al. Relationships Between Retinal Axonal and Neuronal Measures and Global Central Nervous System Pathology in Multiple Sclerosis. *JAMA Neurology*. 2013;70(1):34-43. doi:10.1001/jamaneurol.2013.573

3. Martinez-Lapiscina EH, Arnow S, Wilson JA, et al. Retinal thickness measured with optical coherence tomography and risk of disability worsening in multiple sclerosis: a cohort study. *The Lancet Neurology*. 2016;15(6):574-584. doi:10.1016/S1474-4422(16)00068-5

4. Chattopadhyay A, Hase CH, Zubizarreta JR. Balancing vs modeling approaches to weighting in practice. *Statistics in Medicine*. 2020;39(24):3227-3254. doi:10.1002/sim.8659

5. Ben-Michael E, Keele L. Using Balancing Weights to Target the Treatment Effect on the Treated when Overlap is Poor. *Epidemiology*. 2023;34(5):637. doi:10.1097/EDE.0000000000001644

6. Vickers AJ, Elkin EB. Decision curve analysis: a novel method for evaluating prediction models. *Med Decis Making*. 2006;26(6):565-574. doi:10.1177/0272989X06295361

7. Vickers AJ, van Calster B, Steyerberg EW. A simple, step-by-step guide to interpreting decision curve analysis. *Diagn Progn Res*. 2019;3:18. doi:10.1186/s41512-019-0064-7

Supplementary Results

Detailed multi-modal outcomes and plasma tau markers

The point estimates of associations between baseline markers and subsequent outcomes were qualitatively similar when we applied a longer prediction time window (*i.e.*, 6 vs 3 months post-baseline). While results lost statistical significance for certain associations due to a smaller sample size (*e.g.*, n=160 vs n=142 for the 6- and 3-month post-baseline assessments of PDDS, respectively), general associations remained consistent. For instance, 1 SD increase in concentration of p-tau217 was independently associated with a 0.16 ([95% CI]=[0.02, 0.30]) point increase of PDDS (**Supplementary Figure 3; Supplementary Table 4**). Likewise, p-tau181 association with normalized ARMSS (beta [95%CI]=0.94 [0.24, 1.64]), walking speed (beta [95%CI]=0.62 [0.07, 1.18]), and manual dexterity (beta [95%CI]=3.00 [0.81, 5.19]) remained significant when modeling biomarkers simultaneously (**Supplementary Figure 3; Supplementary Table 4**). Finally, 1 SD elevation in p-tau217 concentration was independently associated with worse neuroimaging metrics: 3.66μm thinner RNFL thickness, 0.66% greater loss of TBV, and 0.95% greater loss of GMV **(Supplementary Table 4**). Associations between baseline p-tau levels and post-baseline thalamic volume and GCIPL thickness, assessed at both 3 and 6 months, were not statistically significant in both separate and simultaneous models (data not shown).

Supplementary Figures

Supplementary Figure 1. Correlations among biomarkers and biomarker correlations with age, sex, and race and ethnicity, and disease duration in people with MS (N = 160).

A. Distribution and correlations of p-tau181, p-tau217, t-tau, t-tau, NfL, and GFAP in pwMS (N = 160). The diagonal shows kernel density estimates of each biomarker; the upper diagonal shows Spearman correlation coefficients; the lower diagonal shows scatterplots where each point represents one participant. There were significant positive correlations between p-tau181 and p-tau217 (corr=0.69, p<0.01), and p-tau181 and t-tau (corr=0.27, p<0.01). NfL correlated with GFAP (corr=0.39, p<0.01) and were both positively correlated with p-tau217.
B. Biomarker concentrations and age (N = 160). Each point represents one participant; blue lines show locally weighted scatterplot smoothing (LOWESS) with 95% confidence intervals (gray). A nonlinear relation with age was observed for all biomarkers.
C. Biomarker concentrations and sex (N = 160; female = 119, male = 41). Comparison was performed using Wilcoxon rank-sum test. T-tau was significantly higher in females than in males (median concentration: 3.13 vs. 2.16 pg/mL; p<0.01). No significant difference was observed for other markers (p>0.05).
D. Biomarker concentrations and race/ethnicity (N = 160; non-Hispanic White = 144, other = 16). Comparison was performed using Wilcoxon rank-sum test. No significant difference was observed between race/ethnicity groups (p>0.05 for all markers).
E. Biomarker concentrations and disease duration (N = 160; ≤5 years = 49; 5-15 years = 49; >15 years = 62). Comparison was performed using Kruskal-Wallis test. All biomarkers except t-tau showed statistically significant differences across disease duration groups.
Abbreviations: *p-tau181*, phosphorylated tau 181; *p-tau217*, phosphorylated tau 217; *t-tau*, total tau; *GFAP,* glial fibrillary acidic protein; *NfL*, neurofilament light chain.

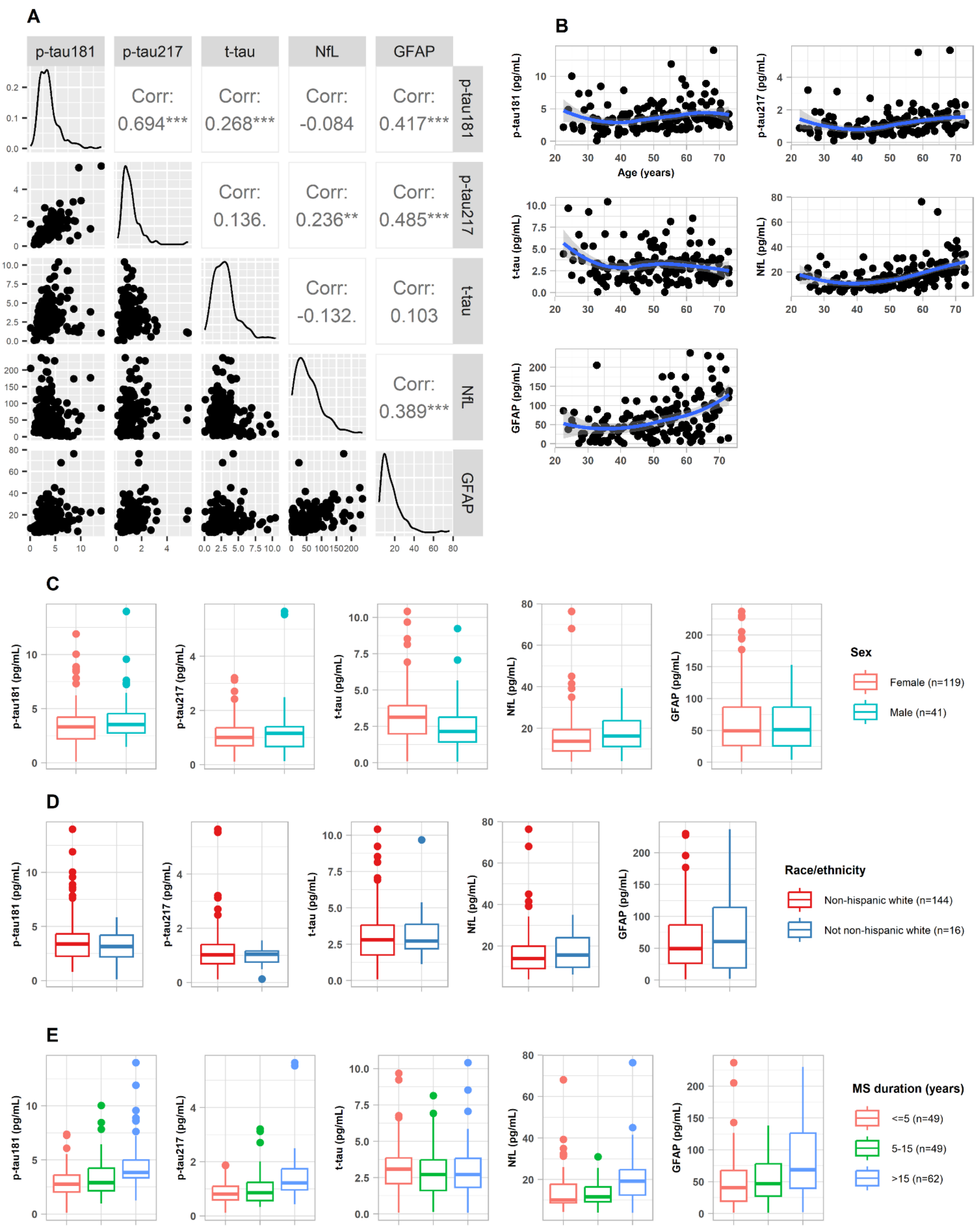
A
p-tau181
p-tau217
t-tau
NfL
GFAP
Corr: 0.694***
Corr: 0.268***
Corr: -0.084
Corr: 0.417***
Corr: 0.136.
Corr: 0.236**
Corr: 0.485***
Corr: -0.132.
Corr: 0.103
Corr: 0.389***
B
p-tau181 (pg/mL)
p-tau217 (pg/mL)
t-tau (pg/mL)
NfL (pg/mL)
GFAP (pg/mL)
Age (years)
C
Sex
Female (n=119)
Male (n=41)
D
Race/ethnicity
Non-hispanic white (n=144)
Not non-hispanic white (n=16)
E
MS duration (years)
<=5 (n=49)
5-15 (n=49)
>15 (n=62)

Supplementary Figure 2. Decision curves for MS subtype prediction models (N = 160).

The value of the net benefit (Y-axis) indicates the benefits of a true positive diagnosis (of MS subtype) minus the harms of a false positive diagnosis for any threshold probability (x-axis). A higher net benefit suggests greater clinical utility of a subtype prediction model at a given threshold probability. For example, at a threshold of 0.5, the probabilities of true PMS among those who were predicted to be PMS by each model (*i.e.*, clinical features alone, clinical features + NfL + GFAP, clinical features + Tau, and clinical features + NfL + GFAP + Tau) are 0%, 4.8%, 5.2%, and 9.9%, respectively. Clinical features included age, sex, race and ethnicity, obesity status, disease duration, and DMT effectiveness. Tau included p-tau181, p-tau217, and t-tau.

Abbreviations: *p-tau181*, phosphorylated tau 181; *p-tau217*, phosphorylated tau 217; *t-tau*, total tau; *GFAP,* glial fibrillary acidic protein; *NfL*, neurofilament light chain.

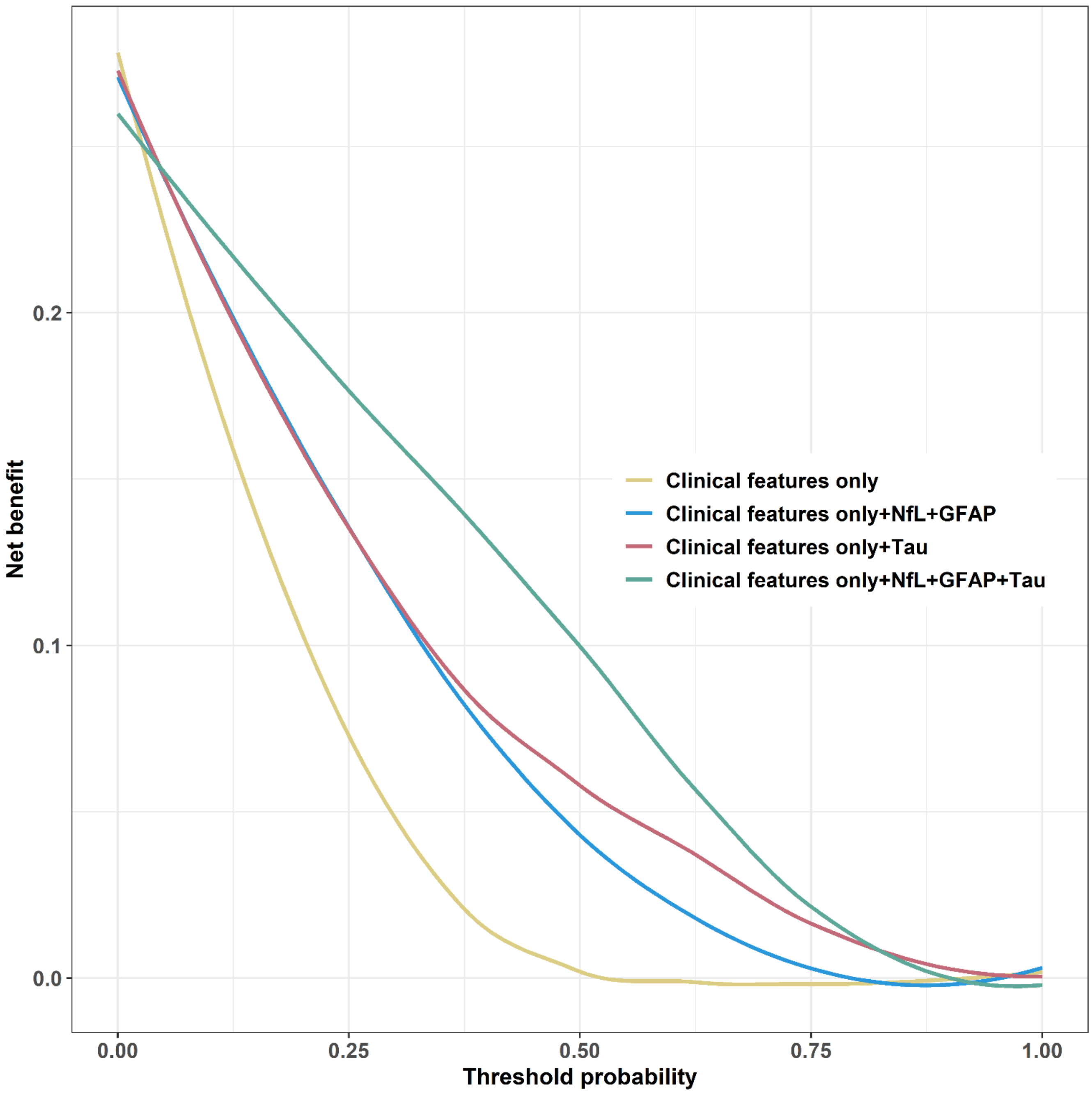

Net benefit
0.2
0.1
0.0
0.00
0.25
0.50
0.75
1.00
Threshold probability
Clinical features only
Clinical features only+NfL+GFAP
Clinical features only+Tau
Clinical features only+NfL+GFAP+Tau

Supplementary Figure 3. Associations between baseline biomarker concentration and multi-modal outcomes 6 months after baseline using generalized equation estimation models.

A. Patient Determined Disease Steps (PDDS); n = 142.
B. Normalized age-related MS severity (ARMSS); n = 66.
C. Timed 25-foot walk test (T25FW); n = 84.
D. Nine-hole peg test (9-HPT) (n = 160); n = 82.
E. Symbol digit modalities test (SDMT) correct score; n = 77.
F. Retinal nerve fiber layer (RNFL) thickness; n = 71.
G. Percent total brain volume loss; n = 57.
H. Percent gray matter volume loss; n = 57.

Generalized equation estimation models were used to estimate associations between biomarkers and repeatedly measured outcomes during follow-up. Models were adjusted for age, sex, race and ethnicity, disease duration, obesity status, MS subtype, baseline PDDS, DMT effectiveness, and 1-year relapse history. Estimates and corresponding 95% CI for a 1 SD increase in each marker concentration are displayed. The red dashed line represents a null association of Beta=0. In "separate" models, each biomarker was separately entered. The estimates are the change in the outcome per 1 SD increase in the biomarker value. In the "simultaneous" models, all biomarkers were simultaneously entered. The estimates are the change in the outcome per 1 SD increase in the biomarker value independent of other markers. The point estimates of associations between baseline markers and subsequent outcomes were qualitatively similar when we applied a longer prediction time window (*i.e.*, 6 vs 3 months post-baseline). While results lost statistical significance for certain associations due to a smaller sample size, general associations remained consistent.

Abbreviations: *p-tau181*, phosphorylated tau 181; *p-tau217*, phosphorylated tau 217; *t-tau*, total tau; *GFAP,* glial fibrillary acidic protein; *NfL*, neurofilament light chain

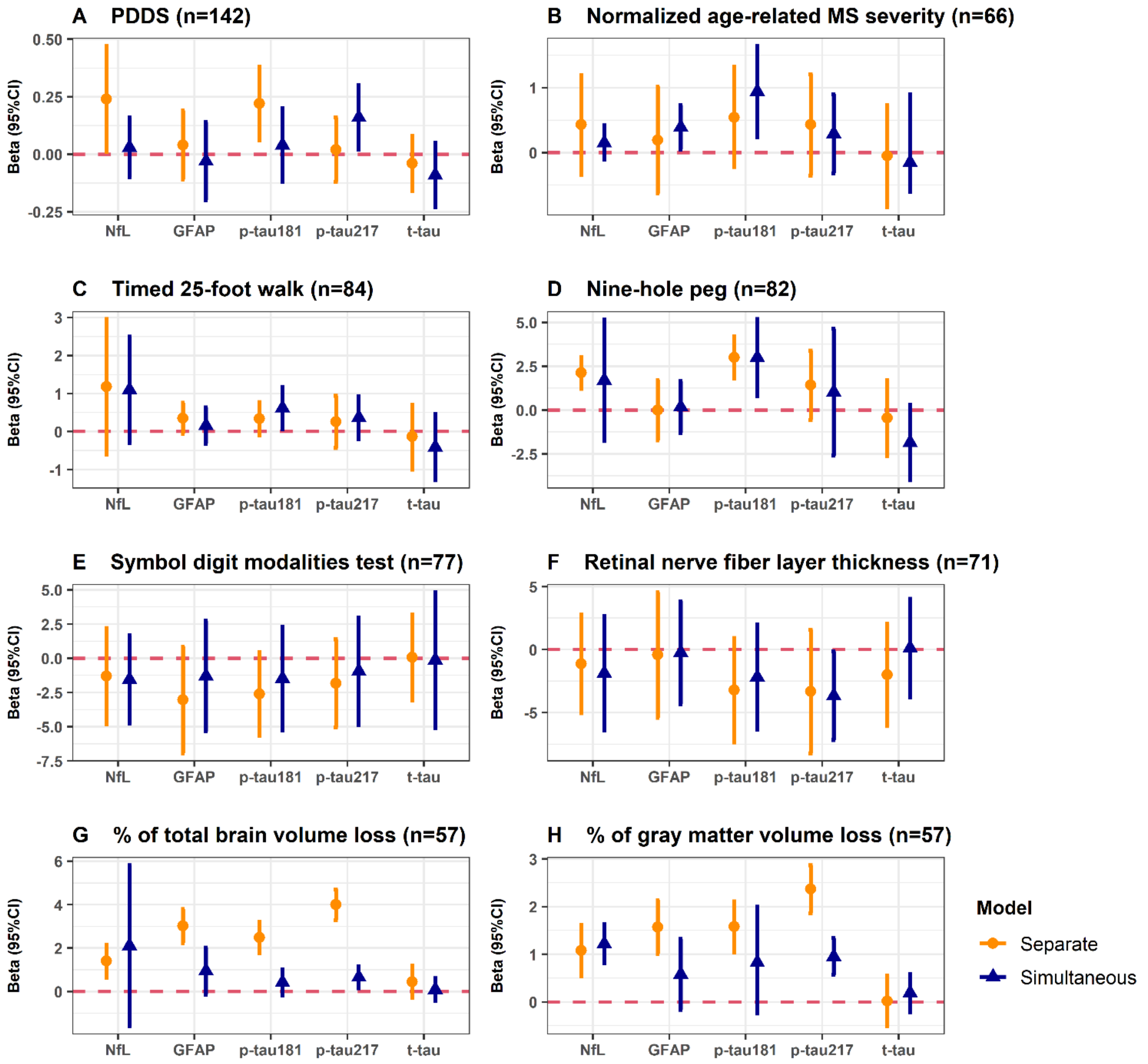
A PDDS (n=142)
B Normalized age-related MS severity (n=66)
C Timed 25-foot walk (n=84)
D Nine-hole peg (n=82)
E Symbol digit modalities test (n=77)
F Retinal nerve fiber layer thickness (n=71)
G % of total brain volume loss (n=57)
H % of gray matter volume loss (n=57)
Beta (95%CI)
NfL
GFAP
p-tau181
p-tau217
t-tau
Model
Separate
Simultaneous

Supplementary Tables

Supplementary Table 1. Performance for MS subtype prediction models using the area under the curve analysis and the decision curve analysis

Predictors	Area under the curve analysis [c]	Decision curve analysis		
		Threshold = 0.25	Threshold = 0.50	Threshold = 0.75
**Set 1**: Clinical features alone [a]	0.64 (0.54, 0.74)	0.08	0	0
**Set 2**: Set 1 +NfL+GFAP	0.82 (0.75, 0.86)	0.14	0.05	0.01
**Set 3**: Set 1 +Tau [b]	0.83 (0.76, 0.89)	0.14	0.06	0.02
**Set 4**: All	0.89 (0.84, 0.94)	0.18	0.10	0.02

Note: Area under the curve analysis evaluated model discrimination as estimated by area under the curve and corresponding 95% CI. Decision curve analysis evaluated the overall clinical utility as estimated by net benefit. See Supplementary Methods. To interpret the decision curve analysis, considering pwMS with a predicted progressive multiple sclerosis (PMS) risk of 0.5 (*i.e.,* at 50% probability threshold, 50% chance to be PMS vs relapsing-remitting multiple sclerosis), incorporation of tau biomarkers added a ~5% higher net benefit than the prediction model containing clinical features, GFAP and NFL (set 2: 0.05; set 4: 0.10).

[a] Clinical features included age, sex, race and ethnicity, obesity status, disease duration, and DMT effectiveness.
[b] Tau markers included p-tau181, p-tau217, and t-tau.
[c] 95%CI from the DeLong test.

Abbreviations: *p-tau181*, phosphorylated tau 181; p-tau217, phosphorylated tau 217; *t-tau*, total tau; *GFAP,* glial fibrillary acidic protein; *NfL*, neurofilament light chain; *DMT*, disease-modifying therapy.

Supplementary Table 2. Model metrics of MS subtype classification

Predictors	Accuracy	F1 score	Sensitivity	Specificity	Positive predictive value	Negative predictive value
**Set 1**: Clinical features alone [a]	0.53	0.77	0.25	0.95	0.52	0.73
**Set 2**: Set 1 +NfL+GFAP	0.74	0.81	0.35	0.79	0.73	0.84
**Set 3**: Set 1 +Tau [b]	0.70	0.81	0.38	0.85	0.78	0.82
**Set 4**: All	0.81	0.85	0.52	0.89	0.82	0.87

Note: Model metrics evaluating the ability to distinguish individuals of progressive MS from relapsing-remitting MS. 5-fold cross-validation with 500 repetitions was used to calculate accuracy, F1 score, sensitivity, specificity, positive and negative predictive values.

[a] Clinical features included age, sex, race and ethnicity, obesity status, disease duration, and DMT effectiveness.
[b] Tau markers included p-tau181, p-tau217, and t-tau.

Abbreviations: *p-tau181*, phosphorylated tau 181; p-tau217, phosphorylated tau 217; *t-tau*, total tau; *GFAP,* glial fibrillary acidic protein; *NfL*, neurofilament light chain; *DMT*, disease-modifying therapy.

Supplementary Table 3. Associations between baseline biomarker concentration and multi-modal outcomes <u>3 months</u> after baseline

Marker	1 SD increase in marker concentration		Relative change compared to 1st tertile		
	Separate estimate (95% CI)[A]	Simultaneous estimate (95% CI)[B]	2nd tertile estimate (95% CI)[C]	3rd tertile estimate (95% CI)[D]	Trend p-value[E]
	**Disability outcome: PDDS (n=160[a])**				
P-tau181	**0.35 (0.14, 0.56)**	**0.31 (0.02, 0.59)**	0.27 (-0.35, 0.94)	**0.73 (0.10, 1.36)**	**0.02**
P-tau217	**0.33 (0.11, 0.56)**	**0.24 (0.03, 0.46)**	0.18 (-0.41, 0.88)	**0.58 (0.05, 1.11)**	**0.01**
T-tau	-0.12 (-0.34, 0.09)	-0.13 (-0.33, 0.07)	0.33 (-0.32, 0.98)	-0.40 (-1.05, 0.24)	0.23
NfL	**0.33 (0.11, 0.55)**	0.23 (-0.02, 0.48)	0.23 (-0.22, 0.68)	**0.77 (0.13, 1.40)**	**0.03**
GFAP	0.01 (-0.27, 0.28)	-0.03 (-0.30, 0.23)	0.43 (-0.19, 0.94)	0.20 (-0.41, 0.82)	0.48
	**Disability outcome: Normalized age-related MS severity score (n=83[b])**				
P-tau181	**0.63 (0.02, 1.24)**	**0.94 (0.36, 1.52)**	0.38 (-0.37, 1.14)	**0.56 (0.38, 0.74)**	0.09
P-tau217	0.49 (-0.05, 1.03)	-0.29 (-0.81, 0.24)	0.39 (-0.55, 1.32)	0.47 (-0.18, 1.12)	0.13
T-tau	-0.05 (-0.84, 0.73)	-0.15 (-0.65, 0.36)	0.30 (-0.48, 1.08)	0.50 (-0.29, 1.29)	0.42
NfL	**0.45 (0.04, 0.86)**	0.15 (-0.17, 0.48)	0.36 (-0.96, 1.69)	0.57 (-0.03, 1.17)	0.21
GFAP	0.19 (-0.13, 0.51)	0.39 (-0.05, 0.83)	0.38 (-1.58, 2.34)	0.73 (-0.50, 1.96)	0.37
	**Functional outcome: Timed 25-foot walk (n=103[c])**				
P-tau181	0.44 (-0.17, 1.05)	**0.68 (0.04, 1.32)**	0.12 (-1.54, 2.51)	0.35 (-1.61, 2.31)	0.13
P-tau217	0.25 (-0.25, 0.75)	0.49 (-0.17, 1.15)	0.22 (-1.84, 2.28)	1.90 (-0.31, 4.11)	0.23
T-tau	-0.51 (-1.20, 0.17)	-0.75 (-1.61, 0.11)	-0.30 (-1.06, 0.46)	1.71 (-2.33,5.75)	0.69
NfL	**0.84 (0.32, 1.36)**	0.86 (-0.20, 1.92)	0.61 (-1.12, 2.34)	0.95 (-1.12, 3.02)	0.18
GFAP	0.28 (-0.38, 0.94)	0.13 (-0.48, 0.73)	-0.11 (-1.70, 2.40)	0.77 (-0.86, 2.39)	0.33
	**Functional outcome: Nine-hole peg (n=99[d])**				
P-tau181	**3.46 (1.37, 5.55)**	**2.49 (0.47, 4.50)**	2.76 (-0.82, 5.40)	**5.70 (3.26, 8.14)**	**0.01**
P-tau217	**1.82 (0.45, 3.20)**	1.66 (-0.73, 4.05)	-1.04 (-2.41,0.33)	2.42 (-2.58, 7.42)	0.34
T-tau	-0.99 (-2.67, 0.69)	-0.27 (-1.66,1.12)	-0.64 (-5.21, 6.91)	1.81 (-3.45, 7.07)	0.44
NfL	**3.19 (0.10, 6.29)**	1.86 (-2.74, 4.99)	1.45 (-1.43,4.33)	**4.14 (2.13, 6.15)**	**<0.01**
GFAP	1.99 (-1.17, 5.14)	0.44 (-1.54, 2.42)	1.60 (-2.13, 5.62)	5.44 (-0.26, 11.15)	0.10
	**Functional outcome: Symbol digit modalities test (n=95[e])**				
P-tau181	-2.75 (-5.66, 0.15)	-1.38 (-2.52, 0.44)	-0.24 (-1.81, 1.33)	**-3.15 (-5.13, -1.17)**	**0.03**
P-tau217	-1.36 (-3.78, 1.05)	-0.88 (-2.72, 0.96)	-1.05 (-3.23, 1.13)	**-2.41 (-4.37, -0.45)**	**<0.01**
T-tau	-0.06 (-2.76, 2.63)	0.01 (-1.71, 1.73)	0.07 (-2.34, 2.48)	-1.13 (-2.95, 0.69)	0.88
NfL	-0.74 (-4.98, 3.51)	-0.94 (-2.49, 0.61)	-0.05 (-3.96, 3.86)	-1.94 (-5.61, 1.73)	0.64
GFAP	-2.16 (-4.93, 0.60)	-1.09 (-3.07, 0.89)	-1.35 (-3.11, 0.41)	-3.90 (-7.84, 0.04)	0.16
	**OCT outcome: Retinal nerve fiber layer thickness (n=84[f])**				
P-tau181	**-4.08 (-6.78, -1.37)**	**-3.31 (-6.78, -0.16)**	**-3.15 (-6.10, -0.10)**	**-5.11 (-9.25, -0.97)**	0.06
P-tau217	**-4.05 (-6.89, -1.21)**	**-4.20 (-7.59, -0.81)**	-2.83 (-6.55, 0.89)	**-7.01 (-10.60, -3.42)**	**<0.01**
T-tau	-2.28 (-4.93, 0.37)	-0.08 (-4.22, 4.06)	-3.06 (-7.20, 1.08)	-0.98 (-4.66, 2.70)	0.45
NfL	-0.55 (-3.25, 2.15)	-1.13 (-4.21, 1.95)	0.03 (-4.50, 4.56)	-1.21 (-4.91, 2.49)	0.83
GFAP	0.88 (-2.03, 3.79)	-0.05 (-4.38, 4.28)	1.20 (-2.60, 5.00)	-2.82 (-7.09, 1.45)	0.44
	**MRI outcome: Percentage of total brain volume loss (n=69[g])**				
P-tau181	**3.32 (2.58, 4.05)**	**0.50 (0.03, 1.07)**	0.78 (-0.10, 1.66)	**0.81 (0.01, 1.61)**	0.13
P-tau217	**4.13 (3.41, 4.85)**	0.29 (-0.23, 0.81)	**1.73 (0.88, 2.58)**	**3.83 (3.04, 4.61)**	**0.03**
T-tau	0.70 (-0.07, 1.46)	0.02 (-0.50, 0.55)	0.38 (-0.31, 1.07)	0.40 (-0.41, 1.21)	0.88
NfL	**2.75 (1.99, 3.50)**	**1.63 (1.13, 2.13)**	0.03 (-0.72, 0.78)	**0.94 (0.14, 1.74)**	**<0.01**

GFAP	**3.35 (2.56, 4.12)**	0.65 (-0.10, 1.40)	**1.51 (0.74, 2.29)**	2.38 (-0.22, 4.98)	0.21
	**MRI outcome: Percentage of gray matter volume loss (n=69[h])**				
P-tau181	**2.12 (1.60, 2.64)**	**0.96 (0.57, 1.34)**	1.40 (-0.30, 3.11)	**1.60 (0.96, 2.24)**	0.21
P-tau217	**2.48 (1.98, 2.98)**	**0.82 (0.43, 1.20)**	0.25 (-0.38, 0.89)	**0.97 (0.33, 1.60)**	**0.02**
T-tau	0.14 (-0.40, 0.68)	0.23 (-0.17, 0.62)	1.50 (-0.41, 3.41)	-0.65 (-1.56, 0.26)	0.96
NfL	**1.77 (1.24, 2.29)**	**1.20 (0.82, 1.58)**	0.22 (-0.35, 0.79)	**2.45 (1.85, 3.05)**	**0.04**
GFAP	**1.76 (1.21, 2.30)**	0.49 (-0.08, 1.06)	**0.80 (0.20, 1.40)**	**1.82 (1.02, 2.63)**	0.11

Note: All models were adjusted for age, sex, race and ethnicity, disease duration, obesity status, MS subtype, baseline PDDS, DMT efficacy, and 1-year relapse history.

[A] Each biomarker was separately entered into the model. The results are the change in the outcome per 1 SD increase in the biomarker concentration.
[B] All markers were simultaneously entered into the model. The results are the change in the outcome per 1 SD increase in the biomarker independent of other markers.
[C] The categorical tertiles of each marker were separately entered into the model. The results are the relative change in the outcome for the 2nd tertile relative to the 1st tertile.
[D] The categorical tertiles of each marker were separately entered into the model. The results are the relative change in the outcome for the 3rd tertile relative to the 1st tertile.
[E] The tertiles of each marker were separately entered into the model as a continuous variable. P-value for trend is from the Wald test.

[a] 160 pwMS had ≥1 PDDS measurement after 3 months from the baseline blood draw.
[b] 83 pwMS had ≥2 EDSS measurement after 3 months from the baseline blood draw.
[c] 103 pwMS had ≥1 timed 25-foot walk test after 3 months from the baseline blood draw.
[d] 99 pwMS had ≥1 Nine-Hole peg test after 3 months from the baseline blood draw.
[e] 95pwMS had ≥1 symbol digit modalities test after 3 months from the baseline blood draw.
[f] 84 pwMS had ≥1 retinal nerve fiber layer thickness measurement after 3 months from the baseline blood draw.
[g] 69 pwMS had ≥1 total brain volume measurement after 3 months from the baseline blood draw.
[h] 69 pwMS had ≥1 gray matter volume measurement after 3 months from the baseline blood draw.

Supplementary Table 4. Associations between baseline biomarker concentration and clinical outcomes <u>6 months</u> after baseline

Marker	1 SD increase in marker concentration		Relative change compared to 1st tertile		
	Separate estimate (95% CI)[A]	Simultaneous estimate (95% CI)[B]	2nd tertile estimate (95% CI)[C]	3rd tertile estimate (95% CI)[D]	Trend p-value[E]
	**Disability outcome: PDDS (n=142[a])**				
P-tau181	**0.22 (0.06, 0.38)**	0.04 (-0.12, 0.20)	0.12 (-0.28, 0.62)	0.25 (-0.10, 0.60)	0.17
P-tau217	0.02 (-0.12, 0.16)	**0.16 (0.02, 0.30)**	0.26 (-0.11, 0.63)	**0.24 (0.05, 0.43)**	0.08
T-tau	-0.04 (-0.16, 0.08)	-0.09 (-0.23, 0.05)	0.11 (-0.47, 0.87)	-0.22 (-0.60, 0.16)	0.41
NfL	**0.24 (0.01, 0.47)**	0.03 (-0.10, 0.16)	0.13 (-0.33, 0.53)	0.37 (-0.14, 0.88)	0.29
GFAP	0.04 (-0.11, 0.19)	-0.03 (-0.20, 0.14)	0.15 (-0.34, 0.64)	0.12 (-0.35, 0.59)	0.60
	**Disability outcome: Normalized age-related MS severity score (n=66[b])**				
P-tau181	0.54 (-0.22, 1.32)	**0.94 (0.24, 1.64)**	0.28 (-0.17, 0.73)	**0.66 (0.36, 0.96)**	0.06
P-tau217	0.43 (-0.35, 1.21)	0.29 (-0.32, 0.90)	0.39 (-0.55, 1.32)	0.47 (-0.18, 1.12)	0.17
T-tau	-0.05 (-0.84, 0.73)	-0.15 (-0.60, 0.90)	-0.05 (-0.48, 0.38)	0.50 (-0.29, 1.29)	0.60
NfL	0.43 (-0.34, 1.19)	0.15 (-0.11, 0.42)	0.36 (-0.96, 1.69)	**0.67 (0.33, 1.00)**	0.05
GFAP	0.19 (-0.63, 1.02)	**0.39 (0.05, 0.73)**	0.38 (-1.58, 2.34)	0.73 (-0.50, 1.96)	0.09
	**Functional outcome: Timed 25-foot walk (n=84[c])**				
P-tau181	0.34 (-0.10, 0.78)	**0.62 (0.07, 1.18)**	0.14 (-1.87, 2.49)	0.44 (-1.17, 2.46)	0.06
P-tau217	0.26 (-0.43, 0.94)	0.37 (-0.19, 0.93)	0.58 (-1.13, 2.44)	0.71 (-1.33, 2.75)	0.36
T-tau	-0.13 (-1.00, 0.71)	-0.41 (-1.28, 0.46)	-0.50 (-1.16, 0.16)	0.02 (-2.13, 2.17)	0.49
NfL	1.18 (-0.60, 2.96)	1.10 (-0.30, 2.50)	0.35 (-1.58, 2.35)	1.93 (-0.08, 2.29)	0.82
GFAP	0.35 (-0.06, 0.76)	0.16 (-0.32, 0.64)	0.08 (-1.78, 2.42)	0.05 (-1.77, 2.45)	0.50
	**Functional outcome: Nine-hole peg (n=82[d])**				
P-tau181	**3.00 (1.79, 4.22)**	**3.00 (0.81, 5.19)**	1.50 (-1.95, 5.85)	**5.36 (1.37, 9.36)**	**<0.01**
P-tau217	1.42 (-0.56, 3.39)	1.02 (-2.60, 4.64)	-1.05 (-6.91,4.80)	2.80 (-3.15,8.75)	0.80
T-tau	-0.45 (-2.61, 1.72)	-1.85 (-4.00,0.30)	0.05 (-5.05, 5.15)	1.71 (-3.00, 6.42)	0.42
NfL	**2.12 (1.22, 3.02)**	1.70 (-1.76, 5.16)	0.17 (-2.76, 3.10)	**6.44 (2.27, 10.62)**	**<0.01**
GFAP	0.00 (-1.72, 1.71)	0.17 (-1.30, 1.65)	1.38 (-3.21, 5.96)	2.20 (-2.59, 6.99)	0.37
	**Functional outcome: Symbol digit modalities test (n=77[e])**				
P-tau181	-2.61 (-5.66, 0.44)	-1.49 (-5.27, 2.29)	-0.35 (-3.82, 3.12)	-3.12 (-7.45, 1.21)	0.19
P-tau217	-1.84 (-5.06, 1.38)	-0.94 (-4.88, 3.00)	-0.17 (-4.05, 3.71)	-2.93 (-6.52, 0.66)	0.07
T-tau	0.07 (-3.08, 3.22)	-0.13 (-5.09, 4.83)	0.03 (-4.05, 4.56)	-0.15 (-3.48, 3.18)	0.87
NfL	-1.31 (-4.84, 2.22)	-1.55 (-4.78, 1.68)	-0.03 (-3.54, 3.48)	-2.19 (-6.48, 2.10)	0.33
GFAP	-3.04 (-6.94, 0.86)	-1.30 (-5.34, 2.74)	-2.22 (-6.43, 1.99)	-2.98 (-6.88, 0.92)	0.52
	**OCT outcome: Retinal nerve fiber layer thickness (n=71[f])**				
P-tau181	-3.21 (-7.35, 0.93)	-2.18 (-6.37, 2.01)	-2.03 (-5.75, 1.69)	-2.88 (-7.16, 1.18)	0.58
P-tau217	-3.33 (-8.23, 1.57)	**-3.66 (-7.17, -0.15)**	-1.15 (-5.13, 2.83)	**-5.03 (-8.70, -1.36)**	**0.03**
T-tau	-2.01 (-6.07, 2.05)	0.13 (-3.77, 4.03)	-2.57 (-6.84, 1.70)	-2.10 (-6.51, 2.31)	0.92
NfL	-1.13 (-5.03, 2.77)	-1.88 (-6.41, 2.65)	-0.04 (-3.80, 3.72)	-1.93 (-5.79, 1.93)	0.31
GFAP	-0.42 (-5.40, 4.56)	-0.25 (-4.33, 3.83)	0.48 (-2.64, 3.60)	-1.07 (-5.17, 3.03)	0.77
	**MRI outcome: Percentage of total brain volume loss (n=57[g])**				
P-tau181	**2.49 (1.77, 3.22)**	0.42 (-0.19, 1.02)	0.36 (-0.41, 1.14)	0.72 (-0.09, 1.52)	0.19
P-tau217	**4.00 (3.29, 4.71)**	**0.66 (0.16, 1.16)**	**2.45 (1.60, 3.31)**	3.38 (-1.21, 7.07)	0.24
T-tau	0.45 (-0.29, 1.19)	0.09 (-0.43, 0.62)	0.28 (-0.33, 0.89)	0.38 (-0.47, 1.22)	0.52
NfL	**1.41 (0.65, 2.16)**	2.11 (-1.60, 5.82)	0.23 (-0.49, 0.94)	**1.64 (0.83, 2.46)**	**<0.01**
GFAP	**3.02 (2.24, 3.80)**	0.95 (-0.13, 2.03)	**1.56 (0.78, 2.35)**	3.19 (-0.09, 6.48)	0.27
	**MRI outcome: Percentage of gray matter volume loss (n=57[h])**				
P-tau181	**1.58 (1.04, 2.11)**	0.83 (-0.24, 2.00)	0.12 (-0.30, 0.54)	**1.03 (0.33, 1.74)**	0.17

P-tau217	**2.37 (1.86, 2.87)**	**0.95 (0.57, 1.34)**	0.66 (-0.01, 1.33)	**1.70 (0.07, 3.33)**	**0.04**
T-tau	0.02 (-0.51, 0.56)	0.19 (-0.21, 0.59)	0.67 (-0.06, 1.40)	-0.04 (-1.99, 1.91)	0.99
NfL	**1.08 (0.54, 1.62)**	**1.22 (0.81, 1.63)**	0.23 (-0.32, 0.79)	**2.92 (2.34, 3.49)**	0.07
GFAP	**1.57 (1.01, 2.14)**	0.58 (-0.16, 1.32)	**0.68 (0.08, 1.28)**	0.69 (-0.07, 1.45)	0.45

Note: All models were adjusted for age, sex, race and ethnicity, disease duration, obesity status, MS subtype, baseline PDDS, DMT efficacy, and 1-year relapse history.

[A] Each biomarker was separately entered into the model. The results are the change in the outcome per 1 SD increase in the biomarker concentration.
[B] All markers were simultaneously entered into the model. The results are the change in the outcome per 1 SD increase in the biomarker independent of other markers.
[C] The categorical tertiles of each marker were separately entered into the model. The results are the relative change in the outcome for the 2nd tertile relative to the 1st tertile.
[D] The categorical tertiles of each marker were separately entered into the model. The results are the relative change in the outcome for the 3rd tertile relative to the 1st tertile.
[E] The tertiles of each marker were separately entered into the model as a continuous variable. P-value for trend is from the Wald test.

[a] 142 pwMS had ≥1 PDDS measurement after 3 months from the baseline blood draw.
[b] 66 pwMS had ≥2 EDSS measurement after 3 months from the baseline blood draw.
[c] 84 pwMS had ≥1 timed 25-foot walk test after 3 months from the baseline blood draw.
[d] 82 pwMS had ≥1 Nine-Hole peg test after 3 months from the baseline blood draw.
[e] 77 pwMS had ≥1 symbol digit modalities test after 3 months from the baseline blood draw.
[f] 71 pwMS had ≥1 retinal nerve fiber layer thickness measurement after 3 months from the baseline blood draw.
[g] 57 pwMS had ≥1 total brain volume measurement after 3 months from the baseline blood draw.
[h] 57 pwMS had ≥1 gray matter volume measurement after 3 months from the baseline blood draw.
